# Prevalence of Pulmonary Embolism and Deep Venous Thromboembolism in Patients With Acute Exacerbation of Chronic Obstructive Pulmonary Disease: A Systematic Review and Meta-Analysis

**DOI:** 10.3389/fcvm.2022.732855

**Published:** 2022-03-09

**Authors:** Weihong Han, Minghang Wang, Yang Xie, Huanrong Ruan, Hulei Zhao, Jiansheng Li

**Affiliations:** ^1^Collaborative Innovation Center for Chinese Medicine and Respiratory Diseases Co-constructed by Henan Province & Education Ministry of P. R. China, Henan University of Chinese Medicine, Zhengzhou, China; ^2^Henan Key Laboratory of Chinese Medicine for Respiratory Disease, Henan University of Chinese Medicine, Zhengzhou, China; ^3^Department of Respiratory Diseases, The First Affiliated Hospital of Henan University of Chinese Medicine, Zhengzhou, China

**Keywords:** acute exacerbation of chronic obstructive pulmonary disease, pulmonary embolism, deep venous thromboembolism, systematic review and meta-analysis, prevalence

## Abstract

**Background:**

Acute exacerbation of chronic obstructive pulmonary disease (AECOPD) increases the risk of pulmonary embolism (PE) and deep venous thrombosis (DVT). AECOPD combined with PE and DVT poses challenges for treatment and management. This necessitates prevention and management to estimate the overall prevalence of PE and DVT among patients with AECOPD and to identify the risk factors.

**Methods:**

We searched the PubMed, Embase, and Cochrane Library databases from their inception to January 9, 2021 and extracted the data from the included studies. The risk of bias was assessed for each study. We separately calculated the prevalence of PE and DVT in patients with AECOPD. Subgroup analysis and meta-regression analyses were performed to determine the sources of heterogeneity. Furthermore, we assessed the publication bias.

**Results:**

The meta-analysis included 20 studies involving 5,854 people. The overall prevalence of PE and DVT among patients with AECOPD was 11% (95% CI: 0.06–0.17) and 9% (95% CI: 0.06–0.12), respectively. Subgroup analysis demonstrated that the prevalence of PE among patients with AECOPD was 12, 2, 7, and 16% in the European, South-East Asia, Western Pacific, and Eastern Mediterranean regions, respectively, and the DVT was 10, 9, 9, and 4%, respectively. The prevalence of PE among patients with AECOPD aged ≥ 70 and <70 years old was 6 and 15%, respectively, and the DVT was 8 and 9%, respectively. The prevalence of PE among patients with AECOPD diagnosed within 48 h and other times (beyond 48 h or not mentioned) was 16 and 6%, respectively, and DVT was 10 and 7%, respectively.

**Conclusion:**

The pooled prevalence of PE and DVT among patients with AECOPD was insignificantly different between the different age groups and the WHO regions. However, the early diagnosis was associated with a higher prevalence of PE. Clinicians and the public need to further improve the awareness of prevention and management for PE and DVT among patients with AECOPD.

**Systematic Review Registration:**

PROSPERO, identifier CRD42021260827.

## Introduction

Chronic obstructive pulmonary disease (COPD) is a common, preventable, and treatable disease characterized by persistent respiratory symptoms and airflow limitation (1). The prevalence of COPD increased by 44.2% to 174.483 million individuals from 1990 to 2015 and the death toll increased by 11.6% (2). Owing to its high prevalence, mortality, and medical costs (3–6), COPD imposes a heavy socioeconomic burden globally and has become a major public health problem. Acute exacerbation of COPD (AECOPD) is defined as an acute worsening of symptoms beyond daily changes, which requires additional treatment (7). The frequency of AECOPD is the major cause of progression and healthcare costs. Most COPD-related deaths occur during acute exacerbations. In addition to infection factors, pollution factors and approximately 30% of the cases are of unknown etiology (8). AECOPD is associated with a prethrombotic state (9). When combined with other factors, such as immobility and infection, AECOPD increases the risk of venous thromboembolism (VTE) in hospitalized patients.

Venous thromboembolism (VTE) includes pulmonary embolism (PE) and deep venous thrombosis (DVT) (10). There are 375,000–425,000 newly diagnosed patients per year, and the conservative cost burden on the healthcare system was $7–10 billion in the United States in 2015 (11). A national cohort study demonstrated that the risk of death for PE and DVT on day 30 was 31 and 3%, respectively (12). In patients with COPD having VTE, the risk of fatality was twice as high as those with normal airflow, and 50% of patients with COPD having stage III/IV died within 3.5 months following the VTE event (13). Moreover, COPD is an independent risk factor of PE. However, the similarity of clinical symptoms between PE and AECOPD makes it difficult to diagnose PE in patients with AECOPD. The failure of clinicians to rapidly initiate anticoagulation therapy worsens the prognosis. Autopsy results also suggested that the prevalence of PE in patients with COPD was approximately 28–51% (14). A meta-analysis indicated that the prevalence of PE in unexplained AECOPD was as high as 29% (15). VTE is a potentially fatal complication in patients with AECOPD, which poses some challenges to their treatment and management. Despite PE and DVT being preventable in several cases, public education appears insufficient. This warrants reliable estimates for PE and DVT among patients with AECOPD and the adoption of timely strategies for its prevention and management. Despite numerous systematic reviews and meta-analyses, there are some variations among different studies and new information has been published. This study aimed to evaluate the pooled prevalence of AECOPD combined with PE and DVT, explore the source of heterogeneity, preliminarily evaluate the pooled prevalence of gender, World Health Organization (WHO) regions, age group, and difference in diagnosis time, and identify related risk factors. We intend to provide a reference for the prevention and management of PE and DVT in patients with AECOPD, besides contributing to public health policy and clinical decision-making.

## Methods

### Literature Search

We conducted a systematic review and meta-analysis following the recommendations of the Cochrane Collaboration and Meta-analysis of Observational Studies in Epidemiology (MOOSE) (16) (Supplementary Table 1).

We searched the PubMed, Embase, and Cochrane Library databases from their inception to January 9, 2021, using search terms consisting of medical subject headings and free-text terms. Detailed search strategies for the different databases were implemented without language restrictions. We attempted to identify eligible articles, and the search terms were modified in different databases. Supplementary Tables 2–4 summarize the search strategy. We manually checked the references of the retrieved articles and previous reviews to identify additional potentially eligible studies.

### Inclusion and Exclusion Criteria

We included the observational studies that reported on the prevalence of PE and/or DVT among patients with AECOPD.

The following article types were excluded: conference abstracts, letters to editors, reviews, meta-analyses, and medical record registrations. We excluded articles containing data with errors and patients diagnosed with non-AECOPD upon our failure to extract information.

### Study Selection and Data Extraction

For the retrieved studies, initially, two individuals (ZHL and RHR) independently screened the titles and abstracts. After excluding irrelevant articles, the rest of the full text of the articles was read. For the final literature included, two individuals independently extracted data (ZHL and RHR). Disagreements were resolved by consultation with a third individual (XY). A data extraction form was prepared for the included studies to extract relevant information, such as authors, the year of publication, geographical region, study design, characteristics of participants, and prevalence.

### Quality Assessment

We assessed the quality of the included studies using a tool modified by Hoy et al. (17). It evaluated the external and internal validity of the literature using 10 items. Each of these items had a score of 1 (yes) or 0 (no), and the total score ranged from 0 to 10. According to the total scores of each study, the risk of bias was classified as low (>8), moderate (6–8), and high (≤5) (18).

### Statistical Analysis

We calculated the raw data from each study as the prevalence, i.e., the number of events that occurred divided by the total sample size. We used the metaprop procedure (19) to perform a meta-analysis of proportions in STATA 14. The procedure is suitable for binomial data and permits the evaluation of the exact binomial and test score-based confidence intervals. It provided suitable strategies for dealing with proportions close to or at the margins where the normal approximation procedures often break down. The metaprop procedure uses a binomial distribution to model the within-study variability or allow the Freeman-Tukey double arcsine transformation to stabilize the variances.

The random-effects model was used to separately estimate the prevalence of PE and DVT among patients with AECOPD. We performed Cochran's Q test and the *I*^2^ test statistic to assess the heterogeneity. An *I*^2^ > 75% was defined as substantial heterogeneity. The subgroup analysis was based on WHO regions, age group (70 years old as the boundary), and diagnosis time. Moreover, we performed a univariate meta-regression to assess the subgroup differences (20).

Sensitivity analysis was performed by excluding each study at a time, which generated the pooled prevalence of remaining studies, compared with the overall pooled prevalence to judge the robustness of our results. Publication bias was evaluated using Begg's test and Egger's test.

Two or more studies have reported on the risk factors for VTE in patients with AECOPD using multivariate models, which was considered for the meta-analysis.

Risk factors for VTE in patients with AECOPD were estimated using odds ratios (ORs) with 95% CIs. We used the random-effects model if the included studies displayed substantial heterogeneity; else, the fixed effects model was used.

For the meta-analysis results, differences were considered statistically significant for *P* < 0.05.

## Results

### Search Results

**Figure 1:**
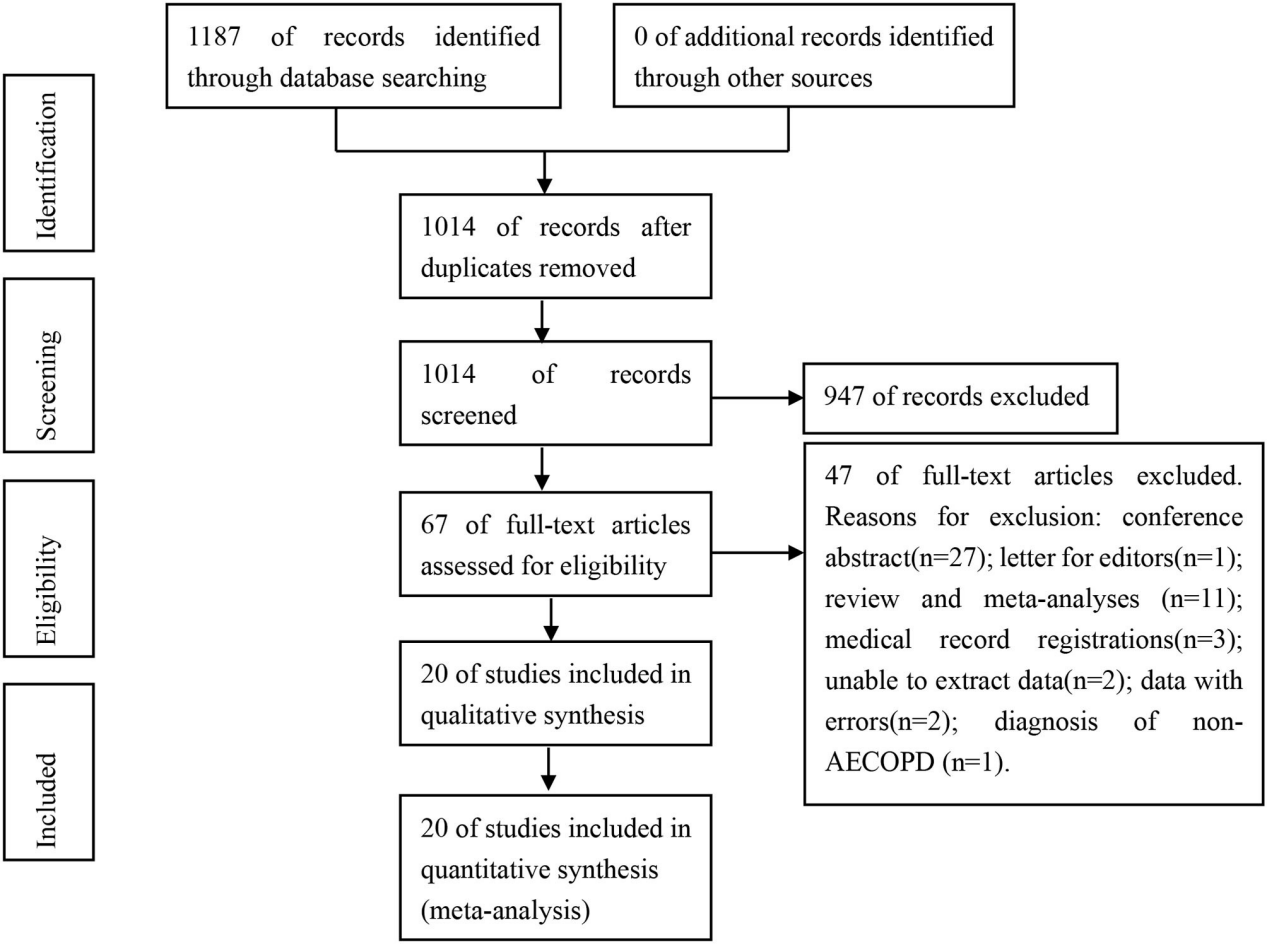
Flow diagram of study selection.

A total of 1,187 related articles were obtained from the initial examination. We excluded 173 duplicate articles, followed by the exclusion of 947 and 47 articles after browsing the title and abstract and full-text analysis, respectively. Eventually, we included 20 articles on the prevalence of PE and DVT in patients with AECOPD (21–40). Of the 20 articles published between 2001 and 2021, only three reported on PE (33–35) and DVT (21, 25, 27) each. Fourteen articles simultaneously reported on PE and DVT (22–24, 26, 28–40). Figure 1 depicts the literature screening process and its result.

### Study Characteristics

The data were principally acquired from 12 countries, namely, China, Turkey, France, Italy, Egypt, India, Iran, Israel, Korea, Singapore, Switzerland, and Tunisia. Four, nine, one, and six countries are in the Eastern Mediterranean region, European region, Southeast Asia region, and Western Pacific region, respectively. This study involved 5,854 people, namely, 3,959 men and 1,795 women, with an average age of 49.3–75.8 years old (one article did not report the number of men and women, whereas one article only reported on the median age). The smallest sample size was 33, and the largest sample size was 1,144. Table 1 summarizes additional characteristics of the included studies.

**Table 1:** Basic characteristics of included studies.

**References**	**Country**	**Study design**	**Diagnosis time**	**Anticoagulant before hospital**	**Sample size**	**Male/female**	**Mean or median age**	**Number of PE**	**Male/female of PE**	**Number of DVT**	**Male/female of DVT**
Pek et al. (21)	Singapore	Prospective	Within 24 h	Exclusion	33	All male	73.8 ± 8.8	#	#	0	0/0
Akgun et al. (22)	Turkey	Prospective	NM	NM	120	82/38	63 ± 11	4	NM	16	NM
Tillie-Leblond et al. (23)	France	Prospective	Within 48 h	NM	197	165/32	60.5 ± 12.1	49	43/6	25	NM
Rutschmann et al. (24)	Switzerland	Cross sectional (prospective)	NM	Exclusion	123	84/39	71 ± 8	4	NM	2	NM
Lessiani et al. (25)	Italy	Prospective	Within 24 h	Exclusion	100	61/39	69 ± 8	#	#	6	3/3
Gunen et al. (26)	Turkey	Prospective	Within 24 h	NM	131	104/27	67.1 ± 10.1	18	NM	14	NM
Duan et al. (27)	China	Prospective	5–7 days after hospital	Exclusion	520	334/186	72 ± 9	#	#	46	30/16
Dutt and Udwadia (28)	India	Prospective	Within 24 h	NM	100	NM	Median 71	2	NM	9	NM
Wang et al. (29)	China	Prospective	Within 24 h	NM	208	158/50	62 ± 12	69	59/10	43	NM
Choi et al. (30)	Korea	Prospective	Within 24 h	Exclusion	103	70/33	71 ± 6	5	2/3	6	NM
Kamel et al. (40)	Egypt	Cross-sectional	Within 24 h	NM	105	all male	49.3 + 8.43	30	NM	11	NM
Liang et al. (31)	China	Retrospective	NM	NM	636	416/220	74.9 ± 9.3	2	NM	92	58/34
Akpinar et al. (32)	Turkey	Prospective	Within 24 h	Exclusion	172	142/30	71.31 ± 9.62	50	38/12	50	NM
Shapira-Rootman et al. (33)	Israel	Prospective	NM	Exclusion	49	35/14	Mean 65.5	9	NM	#	#
Bahloul et al. (34)	Tunisia	Retrospective	Within 48 h*	Inclusion	131	118/13	68.6 ± 9.2	23	21/2	#	#
Davoodi et al. (35)	Iran	Cross-sectional	3 days after hospital	Exclusion	68	38/30	67.75 ± 9.26	5	3/2	#	#
Pang et al. (36)	China	Prospective	7–10 days after hospital	NM	1,144	761/383	72.0 ± 9.1	24	NM	64	NM
Hassen et al. (37)	Tunisia	Prospective cohort study	NM	Exclusion	131	104/27	68 ±13	18	13/5	1	NM
Dentali et al. (38)	Italy	Retrospective multicenter cohort study	NM	Inclusion	1,043	683/360	75.8 ± 9.7	132	66/66	95	NM
Couturaud et al. (39)	France	Cross-sectional study with prospective	Within 48 h	Exclusion	740	466/274	68.2 ± 10.9	44	NM	25	NM

*PE, pulmonary embolism; DVT, deep venous thrombosis; NM, no mention*.

# *not studied*;

* *one case was not diagnosed within 48 h*.

### Results of the Risk of Bias

We evaluated the risk of bias of the included studies, and the major scores of items 1–3 for each study were approximately zero points. Total scores of each study were principally concentrated at six to seven points. The scores for two studies were five points, and those for one study were eight points. According to the scoring criteria, 18 studies were rated with a moderate risk of bias, compared with two studies rated with a high risk of bias (Supplementary Table 5).

### Prevalence of VTE in Patients With AECOPD

**Figure 2:**
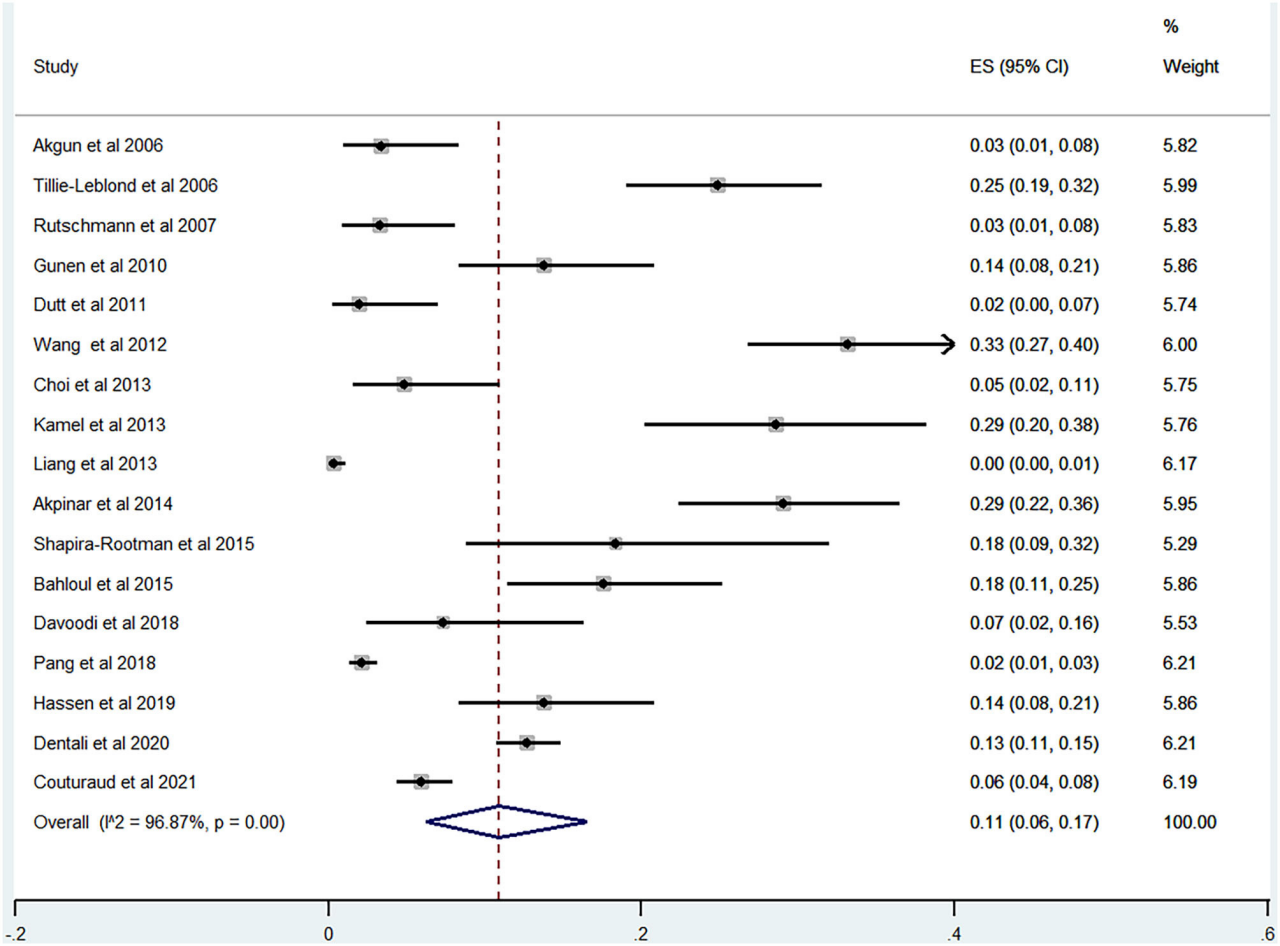
Forest plot of prevalence of PE in AECOPD.

**Figure 3:**
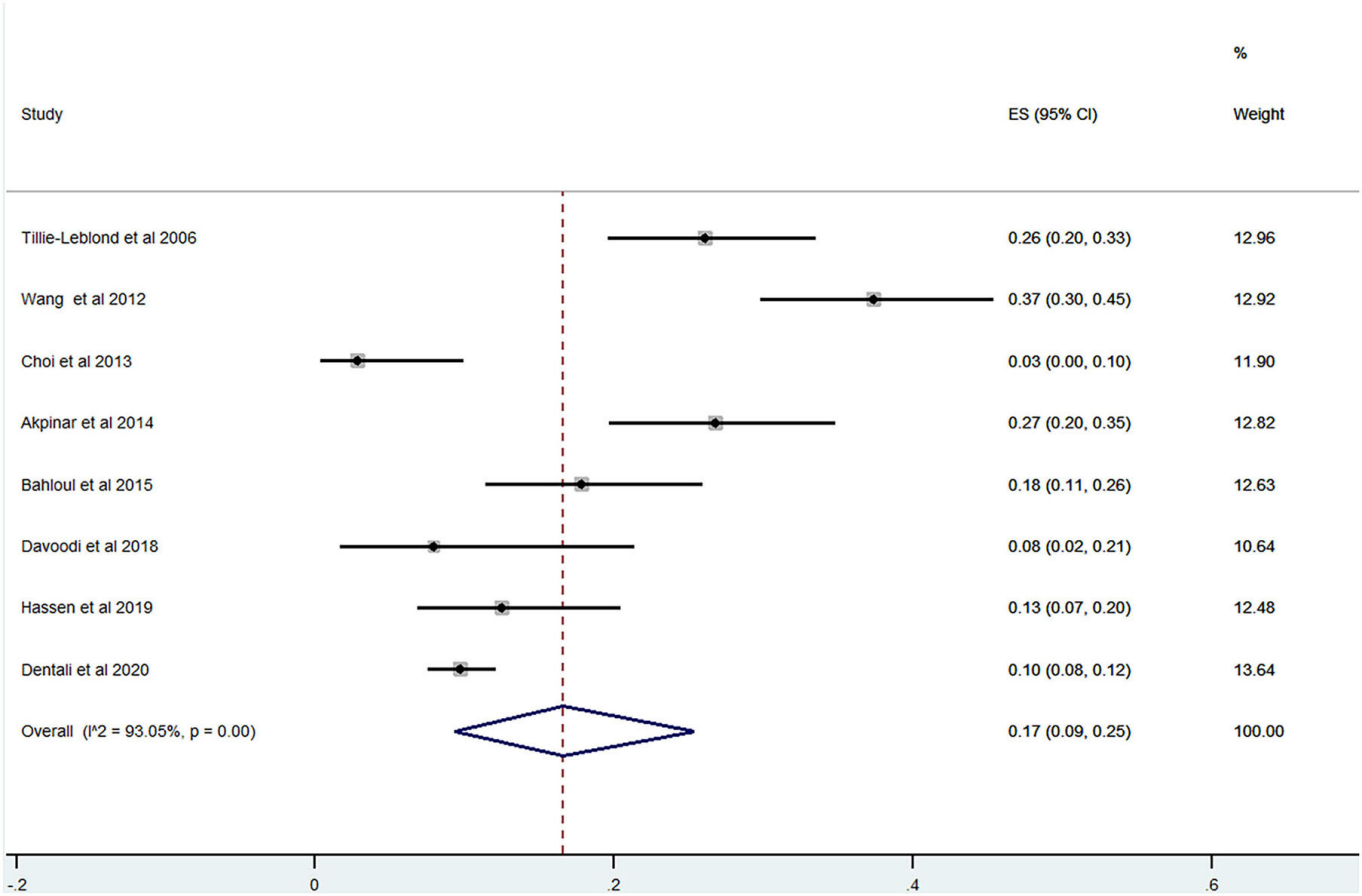
Prevalence of PE among AECOPD in men.

**Figure 4:**
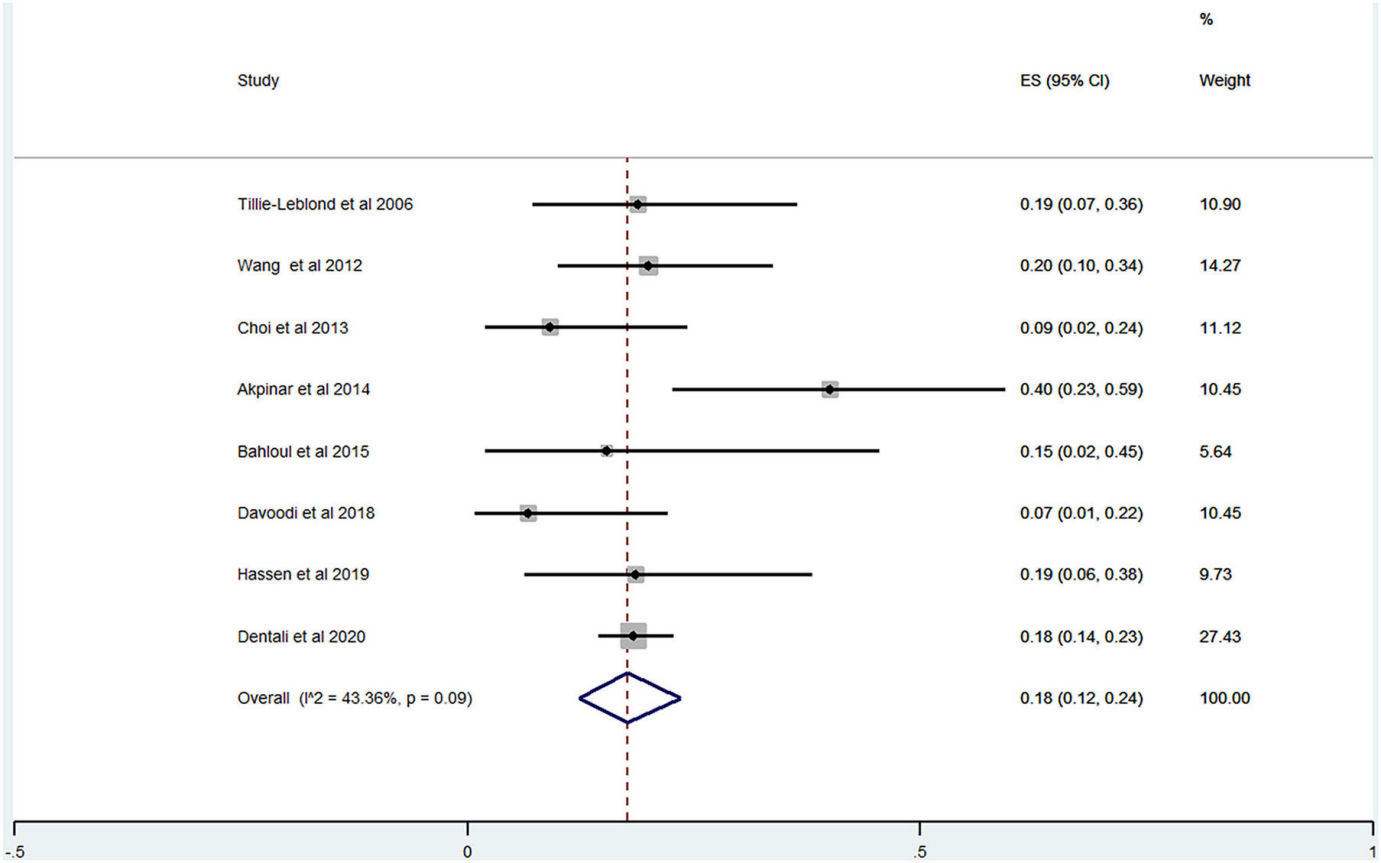
Prevalence of PE among AECOPD in women.

Of the 20 studies, 17 studies (22–24, 26, 28–40) reported the prevalence of PE among patients with AECOPD ranging from 0 to 33% and the estimated pooled prevalence was 11% (95% CI: 0.06–0.17; *I*^2^ = 96.87%) (Figure 2). Of these studies, eight studies (23, 29, 30, 32, 34, 35, 37, 38) reported the proportion of men and women. The pooled prevalence for men and women was 17 (95% CI: 0.09–0.25; *I*^2^ = 93.05%) and 18% (95% CI: 0.12–0.24; *I*^2^ = 43.36%), respectively (Figures 3, 4).

There were no outliers in the results of the sensitivity analysis regarding the prevalence of PE in AECOPD upon excluding one study at a time, which confirmed the stability of our results (Supplementary Figure 1).

**Figure 5:**
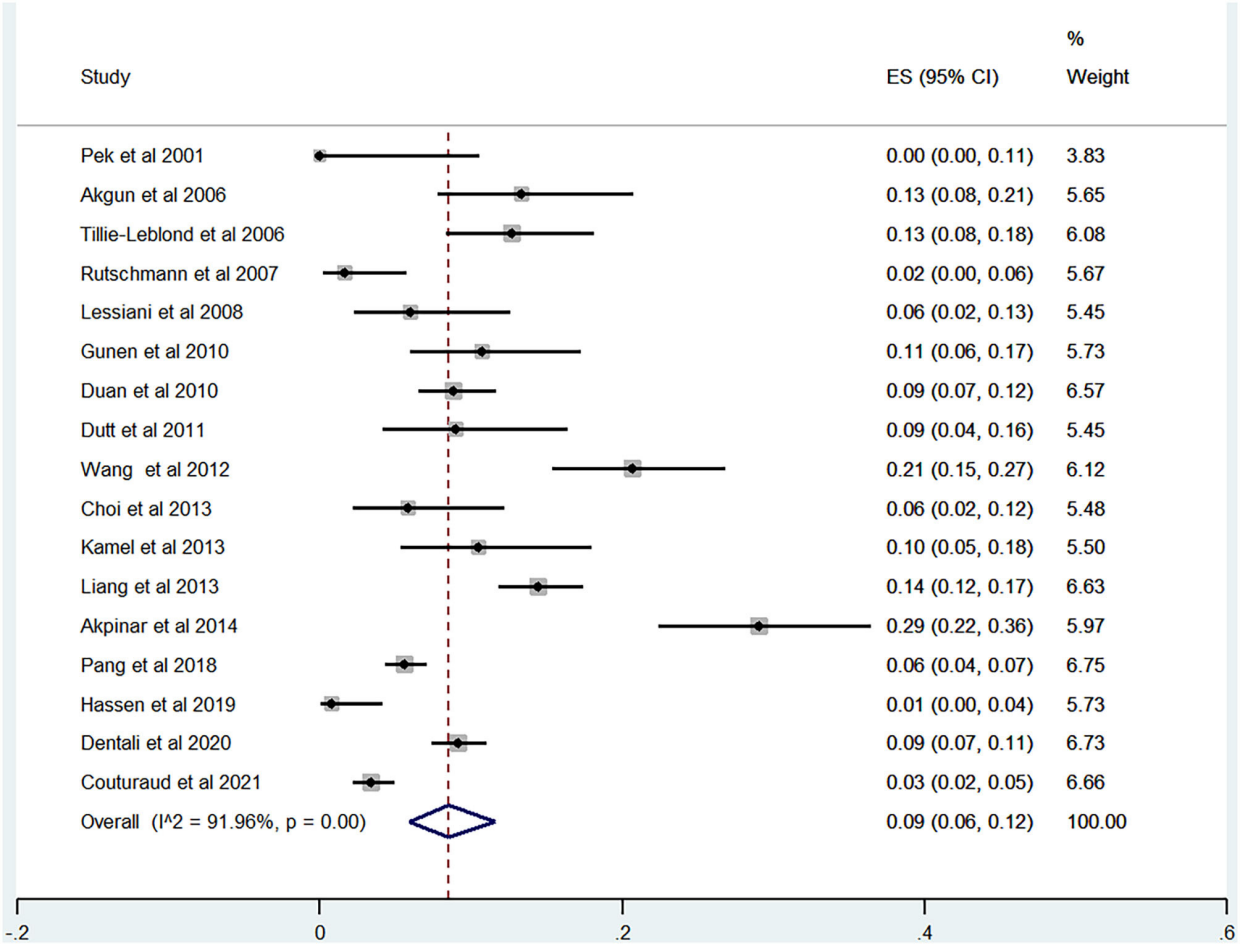
Forest plot of prevalence of DVT in AECOPD.

**Figure 6:**
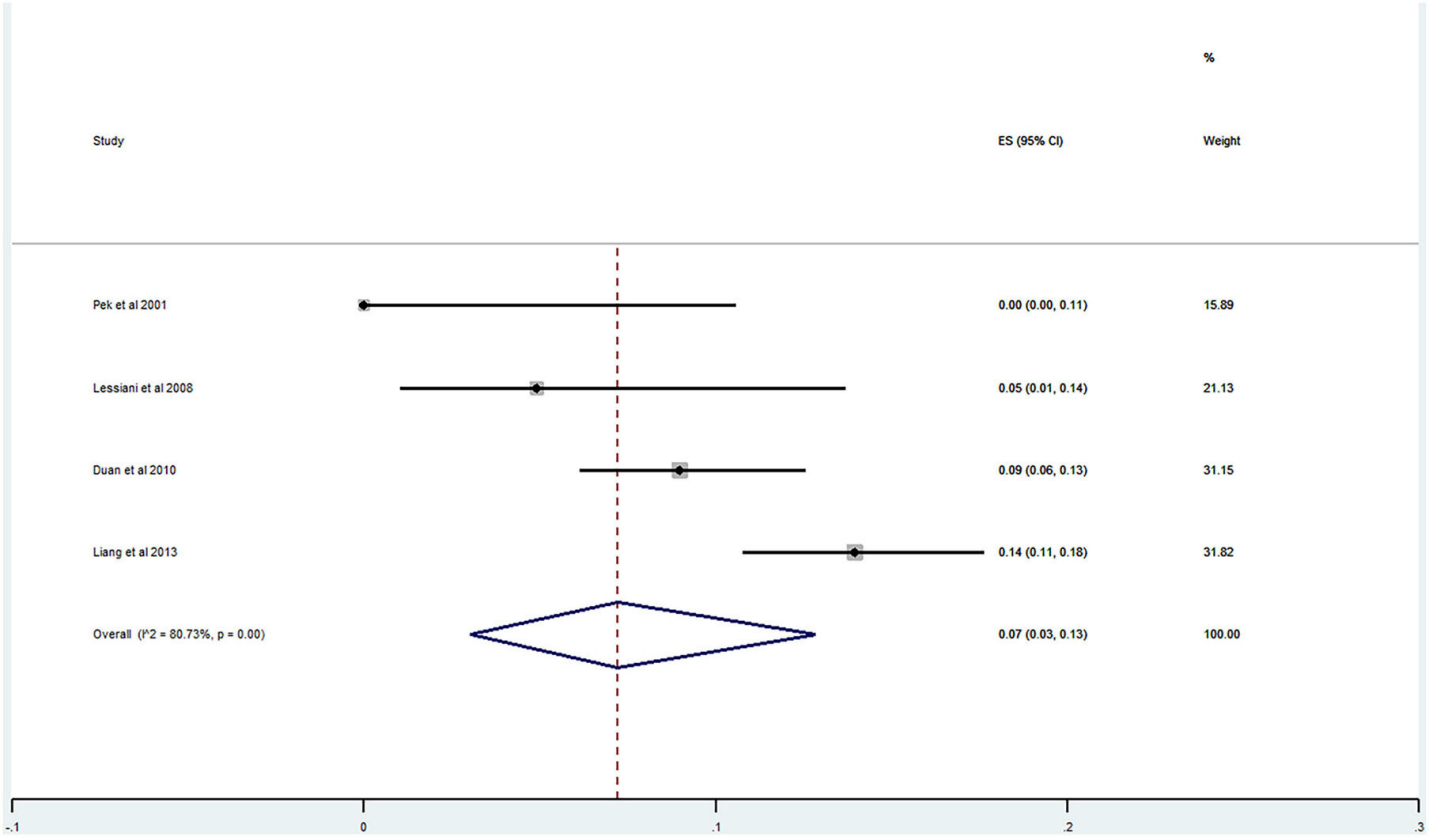
Prevalence of DVT among AECOPD in men.

**Figure 7:**
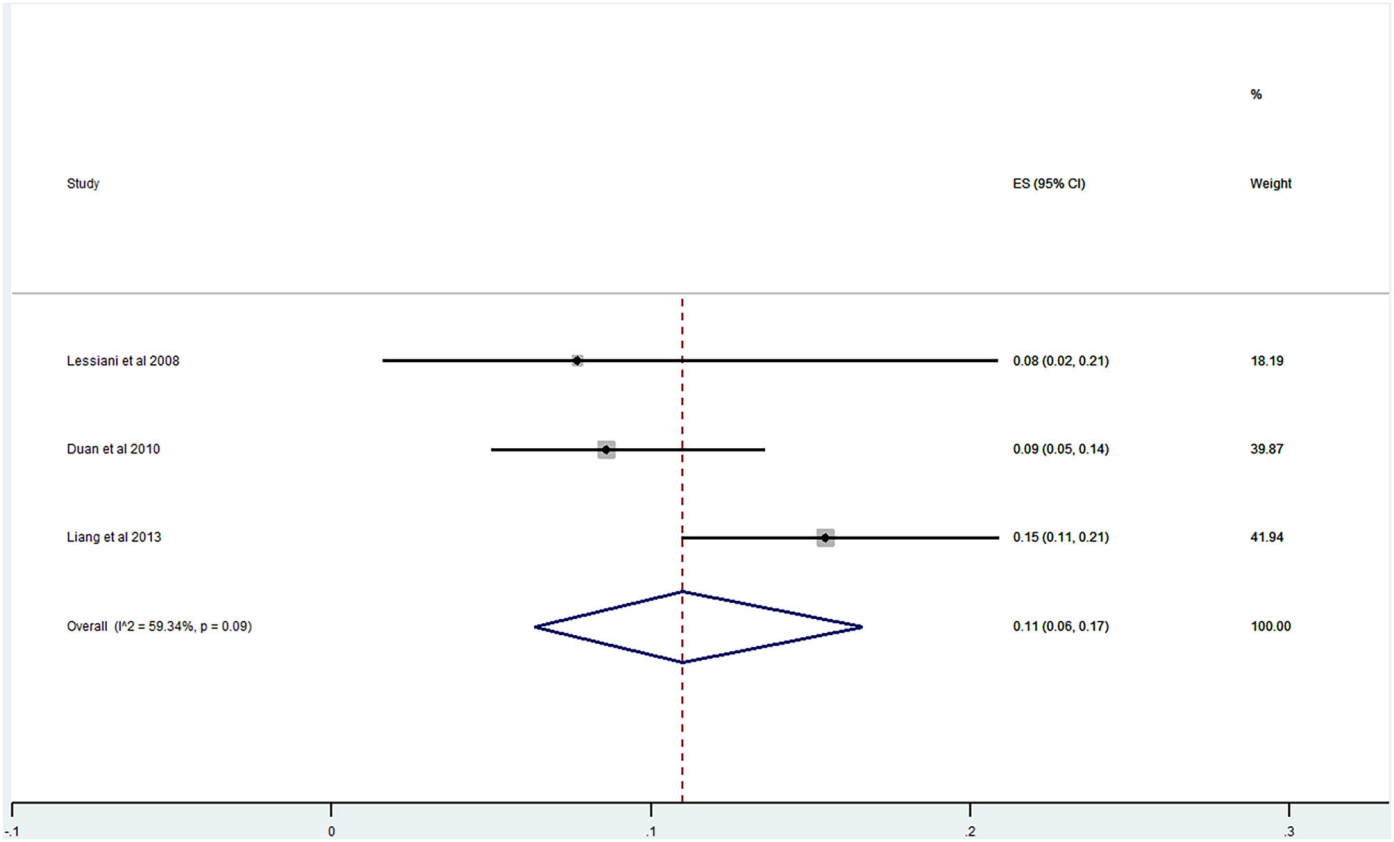
Prevalence of DVT among AECOPD in women.

Of the 20 studies, 17 studies (21–40) reported the prevalence of DVT ranging from 0 to 29%. The pooled prevalence was 9% (95% CI: 0.06–0.12; *I*^2^ = 91.96%) (Figure 5). Three studies (25, 27, 31) simultaneously reported on the proportion of men and women, whereas one study (21) included only men. The pooled prevalence for men and women was 7 (95% CI: 0.03–0.13; *I*^2^ = 80.73%) and 11% (95% CI: 0.06–0.17; *I*^2^ = 59.34%), respectively (Figures 6, 7).

The sensitivity analysis demonstrated that no single study interfered with the results of pooled prevalence by ≥2% on excluding one study at a time, thus indicating good stability (Supplementary Figure 2).

### Subgroup Analysis

The regional prevalence of PE and DVT among patients with AECOPD was 12% (95% CI: 0.07–0.19; *I*^2^ = 93.95%) and 10% (95% CI: 0.05–0.15; *I*^2^ = 93.53%) in the European region, respectively; 7% (95% CI: 0.00–0.19; *I*^2^ = 98.49%) and 9% (95% CI: 0.05–0.14; *I*^2^ = 92.82%) in the Western Pacific region, respectively; 16% (95% CI: 0.09–0.25; *I*^2^ = 79.52%) and 4% (95% CI: 0.02–0.07) in the Eastern Mediterranean region, respectively; and 2% (95% CI: 0.00–0.07) and 9% (95% CI: 0.04–0.16) in the Southeast Asia region, respectively (Table 2).

**Table 2:** Subgroup analysis of the prevalence of VTE in AECOPD and subgroup differences.

**Subgroup**	**PE in AECOPD**			**Univariate meta regression (*P*)**	**DVT in AECOPD**			**Univariate meta regression (*P*)**
	**Heterogeneity test results** **(*I*^**2**^, ***P***)**		**ES%(95%CI)**		**Heterogeneity test results** **(*I*^**2**^, ***P***)**		**ES%(95%CI)**	
**WHO region**								
European Region	93.95%	0.00	12% (95%CI:0.07–0.19)	0.498	93.53%	0.00	10% (95%CI:0.05–0.15)	0.888
South-East Asia Region	-	-	2% (95%CI:0.00–0.07)	0.639	-	-	9% (95%CI:0.04–0.16)	0.916
Western Pacific Region	98.49%	0.00	7% (95%CI:0.00–0.19)	Reference	92.82%	0.00	9% (95%CI:0.05–0.14)	Reference
Eastern Mediterranean Region	79.52%	0.00	16% (95%CI:0.09–0.25)	0.337	-	-	4%(95%CI:0.02–0.07)	0.517
**Age stage**								
≥70years old	97.70%	0.00	6% (95%CI:0.01–0.13)	0.103	92.58%	0.00	8% (95%CI: 0.05–0.12)	0.779
<70 years old	93.99%	0.00	15% (95%CI:0.09–0.23)	Reference	91.94%	0.00	9% (95%CI:0.04–0.15)	Reference
**Diagnosis time**								
within 48 h	95.67%	0.00	16% (95%CI:0.08–0.25)	reference	92.89%	0.00	10% (95%CI: 0.05–0.16)	Reference
NM or beyond 48 h	96.52%	0.00	6% (95%CI:0.02–0.12)	0.034	91.64%	0.00	7% (95%CI: 0.04–0.10)	0.502

*PE, pulmonary embolism; DVT, deep venous thrombosis; VTE, venous thromboembolism; AECOPD, acute exacerbation of chronic obstructive pulmonary disease; NM, no mention*.

In terms of age, the prevalence of PE and DVT among patients with AECOPD aged <70 years old was 15% (95% CI: 0.09–0.23; *I*^2^ = 93.99%) and 9% (95% CI: 0.04–0.15; *I*^2^ = 91.94%) and in those aged ≥70 years old was 6% (95% CI: 0.01–0.13; *I*^2^ = 97.70%) and 8% (95% CI: 0.05–0.12; *I*^2^ = 92.58%), respectively (Table 2).

With respect to the diagnosis time, the prevalence of PE and DVT in patients with AECOPD diagnosed within 48 h was 16% (95% CI: 0.08–0.25; *I*^2^ = 95.67%) and 10% (95% CI: 0.05–0.16; *I*^2^ = 92.89%), respectively. The prevalence of diagnosis beyond 48 h or in those without a diagnosis time was 6% (95% CI: 0.02–0.12; *I*^2^ = 96.52%) and 7% (95% CI: 0.04–0.10; *I*^2^ = 91.64%), respectively (Table 2).

### Subgroup Differences and Meta-Regression

For the prevalence of PE and DVT among patients with AECOPD, subgroup differences were observed in diagnosis time (*P* = 0.034). The results of the subgroup analysis of the age group and WHO regions were insignificantly different (*P* > 0.05) (Table 2).

### Publication Bias

Neither Begg's test nor Egger's test demonstrated significant publication bias in AECOPD with PE (*P* = 1.000 and *P* = 0.095, respectively) and DVT (*P* = 0.773 and *P* = 0.496, respectively) (Supplementary Figures 3–6).

### Risk Factors for VTE in AECOPD

Table 3 summarizes the statistically significant risk factors for VTE in patients with AECOPD in the multivariable model. Seven studies reported on the risk factors of AECOPD combined with VTE (27, 29, 30, 32, 36–38). One study reported on the risk factors for DVT, and five studies reported on the risk factors for PE. The meta-analysis indicated that bedridden/immobility (OR: 2.93; 95% CI: 2.10–4.10; *P* = 0.000) and lower limb asymmetry (OR: 2.51; 95% CI: 1.63–3.86; *P* = 0.000) were the most common risk factors for VTE in AECOPD. However, the pooled analysis of D-dimer levels and age was statistically insignificant (*P* ≥ 0.05) (Table 4).

**Table 3:** Statistically significant risk factors for VTE in patients with AECOPD reported in the included studies.

**References**	**Risk factors**	**OR (95% CI)**
Duan et al. (27)^a^	Current smoking	2.232 (1.214–4.102)
	Pneumonia	2.524 (1.446–4.405)
	Immobility exceeding 3 days	2.916 (1.657–5.132)
	Respiratory failure type II	1.996 (1.153–3.456)
Choi et al. (30)^§^	Plasma D-dimer > 500 ug/l	25.3 (1.4–464.4)
	Absence of symptoms of respiratory infection	31.2 (1.7–562.6)
Wang et al. (29)^§^	Bedridden ≥ 7 days	3.24 (1.56–4.98)
	lower limb asymmetry ≥ 1 cm	2.56 (1.48–3.93)
	DVT	2.31 (1.23–3.58)
Akpinar et al. (32)^§^	obesity	4.97 (1.775–13.931)
	lower limb asymmetry	2.329 (1.127–7.105)
Pang et al. (36)	history of venous thrombosis	16.29 (7.65–34.72)
	Bedridden/immobility ≥3 days	2.20 (1.12–4.31)
	lower limb pain before hospital	unilateral 7.55 (2.41-23.7)
		bilateral 9.91 (2.48-39.6)
	D-dimer	1.36 (1.12– 1.66)
Hassen et al. (37)^§^	Increased sputum volume	0.106 (0.029–0.385)
	Immobilization >7 days	5.024 (1.470–17.170)
	Age ≥70 years old	5.483 (1.269–23.688)
	Invasive mechanical ventilation	3.615 (1.005–13.007)
Dentali et al. (38)^§^	Age	1.03 (1.01–1.06)
	Sex	0.39 (0.25–0.62)
	Hypertension	1.65 (1.03–2.66)
	DVT clinical signs	5.62 (3.11–10.14)
	PCO2 <40 mmHg	1.96 (1.23–3.13)
	Normal chest X-ray	1.95 (1.19–3.18)

a *DVT*;

§ *PE*.

*PE, pulmonary embolism; DVT, deep venous thrombosis; VTE, venous thromboembolism; AECOPD, acute exacerbation of chronic obstructive pulmonary disease*.

**Table 4:** Meta-analysis of risk factors for VTE in patients with AECOPD.

**Risk factors**	**Studies**	**Heterogeneity test**		**Effect model**	**OR (95%CI)**	***P* value**
		***I* ^2^**	***P* value**			
Bedridden/immobility	4	0.0%	0.671	Fixed effect model	2.93 (2.10–4.10)	0.000
D-dimer	2	74.2%	0.049	Random effect model	4.04 (0.25–64.44)	0.323
Lower limb asymmetry	2	0.0%	0.859	Fixed effect model	2.51 (1.63–3.86)	0.000
Age	2	80.1%	0.025	Random effects model	2.01 (0.40–10.02)	0.394

*VTE, venous thromboembolism; AECOPD, acute exacerbation of chronic obstructive pulmonary disease*.

### Location of VTE in AECOPD

Ten studies reported on the location characteristics of VTE, of which five studies simultaneously reported on the location of PE and DVT, whereas others reported only the location of PE or DVT. Table 5 summarizes the location characteristics of VTE. PE was principally located in central (39%), segmental (35%), and subsegmental (26%) regions. DVT occurred predominantly in the proximal vein (36%) and the distal vein (64%) (Tables 6, 7).

**Table 5:** Location characteristics of VTE included in the study.

**Inclusion study**	**VTE**	**Cases**	**Location**
Akgun et al. (22)	DVT	16	Right common femoral vein, three cases; left common femoral vein, two cases;
			Right superficial femoral vein, four cases; left superficial femoral vein, two cases;
			Right popliteal vein, two cases; and between superficial femoral vein and popliteal vein, three cases (two in the right and one in the left)
Tillie-Leblond et al. (23)^a^	PE	43	Central, 20 cases; segmental, 21 cases; and isolated subsegmental, two cases
Rutschmann et al. (24)	PE	4	Lobar, three cases; subsegmental, one case
	DVT	2	Proximal, two cases
Lessiani et al. (25)	DVT	6	Proximal (femoro-popliteal), three cases; distal, three cases
Gunen et al. (26)	PE	18	Central, nine cases; segmental, five cases; subsegmental four cases or bilateral, nine cases; and unilateral, nine cases (right sided alone, seven cases; left sided alone, two cases)
	DVT	14	Bilateral, one case; unilateral, 13 cases (right, six cases; left, seven cases)
Duan et al. (27)	DVT	46	Proximal, 19 cases; distal, 27 cases
Dutt and Udwadia (28)	DVT	9	Bilateral, one case; unilateral, eight cases
Choi et al. (30)	PE	5	Central, five cases (right or left main pulmonary artery, four cases or interlobato-lobar pulmonary artery, one case)
	DVT	6	Proximal, four cases; distal, two cases
Akpinar et al. (32)	PE	50	Main pulmonary artery, 10 cases; segmental, eight cases; and subsegmental, 32 cases or bilateral, five cases; unilateral, 45 cases
	DVT	50	Proximal, 10 cases; distal, 40 cases (distal deep vein, 18 cases; distal superficial vein, 22 cases)
Couturaud et al. (39)	PE	44	Pulmonary trunk, three cases; lobar PE, 14 cases; segmental PE, 24 cases; isolated subsegmental PE, one case; and multiple subsegmental PE, two cases
	DVT	25	Proximal, 10 cases; distal, 15 cases

a *CTA in patients initially referred for suspected PE*.

*PE, pulmonary embolism; DVT, deep venous thrombosis; VTE, venous thromboembolism*.

**Table 6:** Frequency of PE location.

**PE location**	**Cases**	**Percent (%)**
Central	64	39%
Segmental	58	35%
Subsegmental	42	26%
Total	164	100%

*PE, pulmonary embolism*.

**Table 7:** Frequency of DVT location.

**DVT location**	**Cases**	**Percent (%)**
Proximal	48	36%
Distal	87	64%
Total	135	100%

*DVT, deep venous thrombosis*.

## Discussion

Our systematic review and meta-analysis demonstrated that the overall prevalence of PE and DVT among patients with AECOPD was 11% and 9%, respectively, lower than other comorbidities, such as depressive symptoms and pulmonary hypertension in COPD (41, 42). The prevalence was also lower than previously reported results of systematic reviews and meta-analyses [19.9% and 12.4% (43) vs. 16.1% and 10.5%, respectively (44)]. Similar to previous systematic reviews and meta-analyses, we also did not limit the use of anticoagulants before admission. We included some studies that used anticoagulants for any reason before admission and some that did not clearly describe the use of anticoagulants. Moreover, there was a possibility of preventive anticoagulation following admission. Hence, we could not exclude the influence of anticoagulation therapy in reducing the prevalence of VTE in patients with AECOPD. The use of anticoagulants in such patients can reduce the prevalence of DVT and the risk of thrombosis in patients with AECOPD (45, 46). Furthermore, they are more sensitive to warfarin (47). Therefore, we are not sure whether the low prevalence of DVT and PE in patients with AECOPD is due to a generally low prevalence or whether preventive measures are better. However, few randomized controlled trials have evaluated the efficacy of pharmacological prophylaxis in reducing the risk of PE and DVT in patients with AECOPD. In other words, some clinicians do not pay sufficient attention to it.

Different prevalence of PE and DVT among patients with AECOPD may be related to the varied aspects evaluated in this study, compared with previous ones. First, different studies have dissimilar focuses. Second, the databases searched by different studies had diverse search terms and strategies. Third, the inclusion and exclusion criteria were inconsistent. Thus, the above-mentioned factors may lead to a varied amount of literature that meets the requirements in a similar search period. Recently published meta-analyses of AECOPD combined with PE reported a prevalence of 17.2 and 12.9%, respectively (48, 49). We included 20 studies involving 5,854 people, higher than those included in the two recently published papers. Owing to the large sample size, our results may have higher reliability. We also performed a sensitivity analysis of the prevalence of AECOPD combined with PE and DVT, which demonstrated good stability.

It is important to understand the prevalence of PE and DVT between different sexes in patients with AECOPD in terms of epidemiology. A study demonstrated that different genders caused variations in the phenotype, symptom burden, and complications (50). Men with COPD have a higher prevalence of cardiovascular disease and diabetes, whereas women have a higher prevalence of anxiety or depression (51). In our study, the prevalence of PE among men and women with AECOPD was 17% and 18% and the prevalence of DVT between genders was 7 and 11%, respectively. Other relevant systematic reviews and meta-analyses on the prevalence of PE and DVT among patients with AECOPD did not explore gender differences. The overall prevalence of PE and DVT in different sexes with AECOPD is unknown. COPD combined with DVT is principally observed in hospitalized elderly men (52). COPD prevalence in women is similar to or higher than that in men. However, biases while diagnosing COPD in women may cause an underestimation of the number of COPD cases in women (53). This phenomenon may indirectly reduce the detection of PE and DVT in women with AECOPD.

Results of the subgroup analysis demonstrated the varied prevalence of PE and DVT among patients with AECOPD in WHO regions. However, there was no statistically significant difference (*P* > 0.05) between the subgroups, i.e., the region may not be the source of heterogeneity. Data in our study were only acquired from partly countries in WHO regions; therefore, we could not obtain data from other countries. Fu et al. also conducted a subgroup analysis on the regions; however, the results were different owing to different included studies and regional divisions (48). The incidence and prevalence of VTE and COPD differ among different regions and races (54–56). Moreover, the prevalence of PE varies across different regions of a country (57, 58). Therefore, the prevalence of PE and DVT in patients with AECOPD may differ across regions. However, this phenomenon contradicted our findings. We should update the prevalence across different regions with an increase in the number of related studies. Overall, the degree of pharmacological prophylaxis for preventive anticoagulation against PE and DVT may differ among regions. For instance, a quarter of patients at risk of VTE did not receive prophylaxis in Africa (59).

The subgroup analysis results also illustrated that age was not the source of heterogeneity in the prevalence of PE and DVT (*P* > 0.05) on using 70-year-old patients as the boundary. The age-related trend of the prevalence of PE among patients with AECOPD was inconsistent with that of the prevalence of VTE and COPD. Age is a risk factor for VTE, with a high incidence in patients aged ≥ 70 years old (60, 61). For women, the risk of developing COPD increased by 4.7 aged 61–70 years old and 14.5 after 80 years old (62). Moreover, the mortality increased with age (63). Currently, there are few studies on the prevalence of PE and DVT among patients with AECOPD, particularly on the relationship between prevalence and age. Further research is warranted to explain this age-specific shift in the burden of disability.

The diagnosis time was the source of heterogeneity. The prevalence of PE in patients with AECOPD diagnosed within 48 h was higher than those with other diagnostic times (16 vs. 6%, *P* < 0.05). However, different diagnostic times exerted little effect on DVT in our study. According to GOLD guidelines, the risk of PE and DVT increases with the deterioration of COPD, thus necessitating preventive measures of anticoagulation (1). We stratified the prevalence by the diagnosis time, which partially avoided the effect of preventive anticoagulation on PE and DVT during hospitalization. However, this study is only a preliminary report. Some studies did not mention the specific diagnosis time, which may exert an impact on the results. Early diagnosis, detection, and timely treatment of PE can reduce the risk of this disease.

The pooled analysis demonstrated that bedridden/immobility and lower limb asymmetry were the most common independent risk factors for VTE among patients with AECOPD. A recent qualitative analysis reported that immobilization, higher D-dimer level, lower limb edema, DVT, and older age were the dependent predictors (49). In this study, D-dimer and age were not risk factors for VTE in patients with AECOPD. The reasons for differences between the two results are principally attributed to different methods of data extraction and data processing. Moreover, in our studies, the number of studies with risk factors related to D-dimer and age is small, and the confidence interval is large. The incidence of VTE increases with age, and functional impairment is an age-specific risk factor for VTE (64). D-dimer is the expression of endogenous fibrinolytic activity. D-dimer thresholds can determine the need for anticoagulation for patients with suspected PE. Moreover, they distinguish between patients at a high risk of clinical deterioration and nondeterioration (65). Akpinar et al. revealed that D-dimer cut-off level for excluding PE in COPD exacerbation was 0.95 pg/ml (66). However, D-dimer levels of AECOPD combined with VTE vary across studies, thus warranting further research.

Understanding the risk factors of VTE among patients with AECOPD facilitates the early identification of high-risk patients and reduces the risk of VTE. In 2003, Ambrosetti et al. systematically reviewed the prevalence and prevention of AECOPD together with VTE in the MEDLINE database from 1966 to 2003 (67). A global survey demonstrated that the public lacks awareness of PE and DVT. Similarly, they have little information about the related symptoms, signs, and risk factors (68). Recently, Fu et al. also reported the clinical features of AECOPD combined with VTE (48). Furthermore, following anticoagulant therapy, there was no significant difference in the risk of recurrent VTE, compared with those without COPD (69). Thus, understanding the risk factors of AECOPD combined with VTE is a prerequisite for reducing the prevalence and the prevention of VTE.

In this study, most articles simultaneously reported on PE with DVT. However, there are few reports on DVT and PE only. The RIETE registry reported that patients with COPD having PE have an increased risk of PE recurrence and death, and require more effective treatment than those with DVT alone (70). PE is supposedly caused by the movement of blood clots in the venous system. Despite pieces of evidence for *in-situ* pulmonary artery thrombosis, the displacement of venous thrombosis, particularly PE caused by DVT of the lower extremities, warrants attention (71). More than 90% of PE originates from DVT in the lower extremities (72). Approximately 70% of fatal PE cases result from lower extremity thrombosis (73). Approximately, one-third of the patients with DVT have silent PE (74). Therefore, the prevention, screening, and management of DVT can reduce the morbidity and mortality of PE in patients with AECOPD.

This study demonstrated that the location of PE in patients with AECOPD was mainly central, segmental, and subsegmental. The location of DVT among patients with AECOPD was about 64% in the distal vein and the proximal vein was 36%. The REMOTEV registry demonstrated that patients with PE-related DVT had more severe PE. During DVT, the proximal location is significantly associated with the severity of PE (75). In patients with isolated acute DVT and initial symptoms, distal DVT is more common in women than that in men. In contrast, men display a higher proportion of proximal events (76). The location of the thrombus is related to the treatment and management of VTE. Researchers recommend 3-month anticoagulant therapy for patients with proximal DVT or PE (77). Direct oral anticoagulant therapies can reduce the recurrence rate, massive bleeding rate, and mortality of VTE in patients with proximal and distal DVT (78).

The methodological quality assessment of prevalence varies across randomized controlled trials that commonly use recommended risk assessment tools (79). Migliavaca et al. (80) reported on 30 tools related to prevalence studies. Of these tools, eight were specifically designed for studies on the quality assessment of prevalence. We used a tool modified by Hoy et al. (17). The included articles had a moderate risk of bias in our study. Nevertheless, suitable methodological evaluation tools were limited, and scores of the first to third items were 0 in most of the studies. Considering the relatively low prevalence and the lack of large-scale national sampling surveys and censuses, most studies and the evaluation of external effectiveness items are not completely compatible, which may partially affect the risk of bias.

## Limitations

The included studies had high heterogeneity, which was analyzed by the subgroup analysis. The difference in the diagnosis time among the prevalence of PE in patients with COPD was partly revealed sources of heterogeneity. However, we only explored important factors. Unexamined factors, such as the type of living ward, the severity of disease, the severity of COPD, and publication year may have contributed to the heterogeneity. Despite the inclusion of heterogeneous studies, the sensitivity analysis results were stable. Besides no publication bias, the results were relatively reliable. Because of the lack of a suitable methodological evaluation tool, we may have overestimated the risk of bias. Moreover, DVT commonly occurs in the legs; however, the deep veins of the visceral areas, cerebral areas, and arms may also be affected (81). Most studies assessed the lower limbs; CT angiography was performed only on suspecting PE in most studies, which may have led to an underestimation of the prevalence of PE and DVT among patients with AECOPD. Considering the relatively small number of studies that met the inclusion criteria and larger heterogeneity, the results should be carefully interpreted, particularly the findings of the WHO region subgroup, and further research is warranted.

## Conclusion

The prevalence of PE and DVT among patients with AECOPD is relatively low and there was no significant difference between the WHO regions and age groups. Its early diagnosis was associated with a higher prevalence. Bedridden/immobility and lower limb asymmetry are the most common independent risk factors for VTE in patients with AECOPD. Clinicians and the public should improve their awareness of disease prevention and management.

## Data Availability Statement

The raw data supporting the conclusions of this article will be made available by the authors, without undue reservation.

## Author Contributions

JL, MW, and HZ participated in the conception and design of the study. HZ formulated the retrieval strategy and conducted the data retrieval. WH, HZ, YX, and HR participated in data extraction, calculation, or analysis. WH drafted the manuscript. All authors read and modified the content of the manuscript and determined the final manuscript.

## Funding

This study was funded by the National Natural Science Foundation of China (8830116 and 81873278), the Qihuang Scholars Award of the State TCM Academic Leader Program [No. (2018)284], and Zhong-yuan Scholars and Scientists Project [No. (2018)204].

## Conflict of Interest

The authors declare that the research was conducted in the absence of any commercial or financial relationships that could be construed as a potential conflict of interest.

## Publisher's Note

All claims expressed in this article are solely those of the authors and do not necessarily represent those of their affiliated organizations, or those of the publisher, the editors and the reviewers. Any product that may be evaluated in this article, or claim that may be made by its manufacturer, is not guaranteed or endorsed by the publisher.
