# Supplementary material for: Prevalence of Pulmonary Embolism and Deep Venous Thromboembolism in Patients With Acute Exacerbation of Chronic Obstructive Pulmonary Disease: A Systematic Review and Meta-Analysis
Source: Front Cardiovasc Med. 2022 Mar 9;9:732855. doi: 10.3389/fcvm.2022.732855 (PMC8959435; doi:10.3389/fcvm.2022.732855)
Supplement: Supplementary file 1 [file Data_Sheet_1.docx]

Supplementary Material

# Supplementary Tables

**Table 1 Moose checklist**

| **Item No** | **Recommendation** | **Brief description of how the criteria were handled in the meta-analysis** |  |
| --- | --- | --- | --- |
| Reporting of background should include | |  |  |
| 1 | Problem definition | AECOPD is associated with a prethrombotic state, and patients with AECOPD increased risk of VTE in hospitalized. | **√** |
| 2 | Hypothesis statement | The purpose of this study is to evaluate the pooled prevalence of AECOPD combined with PE and DVT, to explore the source of heterogeneity, to preliminarily evaluate the pooled prevalence of gender, WHO regions, age group, and difference in diagnosis time, and to understand related risk factors. We hope to provide a reference for the prevention and management of diseases and contribute to public health policy and clinical decision-making. | **√** |
| 3 | Description of study outcome(s) | Estimate the prevalence of PE and DVT among AECOPD. | **√** |
| 4 | Type of exposure or intervention used |  | **NA** |
| 5 | Type of study designs used | Observational study | **√** |
| 6 | Study population | Patients with AECOPD combined PE and / or DVT | **√** |
| Reporting of search strategy should include | |  |  |
| 7 | Qualifications of searchers (eg, librarians and investigators) | - | **NA** |
| 8 | Search strategy, including time period included in the synthesis and key words | PubMed, Embase, and Cochrane Library were searched from the inception of each database to January 9, 2021: using the search terms consisting of medical subject headings and free text terms. The search strategy is presented in Supplementary table2-4.  key worlds: Acute Exacerbation of Chronic Obstructive Pulmonary Disease, Pulmonary Embolism, Deep Venous Thromboembolism, Systematic Review and Meta-Analysis, Prevalence | **√** |
| 9 | Effort to include all available studies, including contact with authors | We searched the literature comprehensively as far as possible, not only in the database, and references of the retrieved articles and previous reviews were also checked manually to identify additional potentially eligible studies. | **√** |
| 10 | Databases and registries searched | PubMed, Embase, Cochrane Library | **√** |
| 11 | Search software used, name and version, including special features used (eg, explosion) | PubMed, Embase, Cochrane Library | **√** |
| 12 | Use of hand searching (eg, reference lists of obtained articles) | References of the retrieved articles and previous reviews were also manually checked to identify additional potentially eligible studies. | **√** |
| 13 | List of citations located and those excluded, including justification | Details of the literature search process are outlined in the supplementary materials. And these reasons were showed by inclusion exclusion criteria. | **√** |
| 14 | Method of addressing articles published in languages other than English | without language restrictions. | **√** |
| 15 | Method of handling abstracts and unpublished studies | We excluded articles with only abstracts. Seeing flow diagram of study selection for details | **√** |
| 16 | Description of any contact with authors |  | **NA** |
| Reporting of methods should include | |  |  |
| 17 | Description of relevance or appropriateness of studies assembled for assessing the hypothesis to be tested | Detailed inclusion and exclusion criteria were described in the methods section. | **√** |
| 18 | Rationale for the selection and coding of data (eg, sound clinical principles or convenience) | A data extraction form for the included studies was made to extract the relevant information of authors, year of publication, geographical region, study design, characteristics of participants, prevalence and so on. | **√** |
| 19 | Documentation of how data were classified and coded (eg, multiple raters, blinding and interrater reliability) | For the retrieved studies, initially two individuals independently screened the titles and abstracts. If they met inclusion criteria, then the rest of full text of the articles will be read and two individuals independently extracted data. Disagreements were resolved by consultation with the third individual. | **√** |
| 20 | Assessment of confounding (eg, comparability of cases and controls in studies where appropriate) | Subgroup analysis was based on WHO regions, age group and diagnosis time. Univariate meta-regression was performed by to judge subgroup differences. | **√** |
| 21 | Assessment of study quality, including blinding of quality assessors, stratification or regression on possible predictors of study results | We assessed the quality of included studies with a modified tool by Hoy et al | **√** |
| 22 | Assessment of heterogeneity | Heterogeneity was assessed by the Cochran’s Q test and I^2^test statistic. | **√** |
| 23 | Description of statistical methods (eg, complete description of fixed or random effects models, justification of whether the chosen models account for predictors of study results, dose-response models, or cumulative meta-analysis) in sufficient detail to be replicated | We chose the random-effects model to estimate the prevalence of PE and DVT among AECOPD. We used metaprop procedure to perform meta-analysis of proportions in STATA 14. Subgroup analysis, univariate meta-regression and sensitivity analysis was also performed. | **√** |
| 24 | Provision of appropriate tables and graphics | Several tables were used to presented basic characteristics of included studies, related risk factors, location characteristics of VTE included in the study. Subgroup analysis of the prevalence of PE and DVT in AECOPD and difference of subgroup. Several graphs were used to describe the main findings of the analyses and findings. Other results were presented in supplementary materials. | **√** |
| Reporting of results should include | |  |  |
| 25 | Graphic summarizing individual study estimates and overall estimate | We have appended them in the main text. Additional graphs were presented as supplementary material to fully describe the results. | **√** |
| 26 | Table giving descriptive information for each study included | Table 1, Table 2, Table 3, Table 5 and supplementary materials. | **√** |
| 27 | Results of sensitivity testing (eg, subgroup analysis) | Sensitivity analysis was performed by excluding each study at a time, and the results of our study confirmed stability. | **√** |
| 28 | Indication of statistical uncertainty of findings | We did report estimates for the main outcome and 95% CI. | **√** |
| Reporting of discussion should include | |  |  |
| 29 | Quantitative assessment of bias (eg, publication bias) | Descriptions of quantitative assessment of bias are detailed in the methods; results are described in the main text and supplementary materials. | **√** |
| 30 | Justification for exclusion (eg, exclusion of non-English language citations) | The following article types were excluded: conference abstract, letters to editors, reviews, meta-analyses and medical record registrations. Studies were also excluded if we were unable to extract data from the study if the studies contained data with errors and if the studies contained patients with a diagnosis of non-AECOPD. | **√** |
| 31 | Assessment of quality of included studies | We also used a modified tool by Hoy to assess quality of included studies. Descriptions of quantitative assessment of bias are detailed in the methods; results are described in the main text and supplementary materials. | **√** |
| Reporting of conclusions should include | |  |  |
| 32 | Consideration of alternative explanations for observed results | We discussed alternative explanations for our findings and compared with others. | **√** |
| 33 | Generalization of the conclusions (ie, appropriate for the data presented and within the domain of the literature review) | We have addressed the generalization of the conclusions in the discussion. | **√** |
| 34 | Guidelines for future research | Clinical and public still need to further improve their awareness of disease prevention and management. | **√** |
| 35 | Disclosure of funding source | National Natural Science Foundation of China (81830116,81873278); the Qihuang Scholars Award of the State TCM Academic Leader Program (No. (2018)284); Zhong-yuan Scholars and Scientists Project (No. (2018)204). | **√** |

**Supplementary Table 2-4. Search Strategies on the Systematic Review and Meta Analysis**

**Table 2 Search Strategies of Embase**

| #1. 'chronic obstructive lung disease'/exp |
| --- |
| #2. 'chronic obstructive pulmonary disease':ab,ti OR 'chronic obstructive airway disease':ab,ti OR 'chronic obstructive lung disease':ab,ti OR 'chronic airflow obstruction':ab,ti OR copd:ab,ti OR coad:ab,ti OR aecopd:ab,ti OR 'acute exacerbation of chronic obstructive pulmonary disease':ab,ti |
| #3. 'lung embolism'/exp |
| #4. 'pulmonaryembolism':ab,ti OR 'pulmonary thromboembolism':ab,ti |
| #5. 'venous thromboembolism'/exp |
| #6. 'venous thromboembolism':ab,ti |
| #7. 'vein thrombosis'/exp |
| #8. 'deep vein thrombosis'/exp |
| #9. 'venous thrombosis':ab,ti OR phlebothrombosis:ab,ti OR phlebothromboses:ab,ti OR 'deep vein thrombosis':ab,ti OR 'deep venous thrombosis':ab,ti OR 'deep vein thromboses':ab,ti OR 'deep venous thromboses':ab,ti |
| #10. prevalence:ab,ti OR incidence:ab,ti OR 'incidence rate':ab,ti OR occurrence:ab,ti OR 'occurrence rate':ab,ti |
| #11. #1 OR #2 |
| #12. #3 OR #4 OR #5 OR #6 OR #7 OR #8 OR #9 |
| #13. #10 AND #11 AND #12 |

**Table 3 Search Strategies of Cochrane**

| #1 MeSH descriptor: [Pulmonary Disease, Chronic Obstructive] explode all trees |
| --- |
| #2 (Chronic Obstructive Lung Disease):ti,ab,kw OR (Chronic Obstructive Airway Disease):ti,ab,kw OR (COPD):ti,ab,kw OR (Chronic Obstructive Pulmonary Disease):ti,ab,kw OR (COAD):ti,ab,kw |
| #3 (Airflow Obstruction*, Chronic):ti,ab,kw OR (Chronic Airflow Obstruction*):ti,ab,kw OR (Acute exacerbation of chronic obstructive pulmonary disease):ti,ab,kw OR (AECOPD):ti,ab,kw |
| #4 MeSH descriptor: [Pulmonary Embolism] explode all trees |
| #5(Embolism*, Pulmonary):ti,ab,kw OR (Pulmonary Embolisms):ti,ab,kw OR (Pulmonary Thromboembolism*):ti,ab,kw OR (Thromboembolism*, Pulmonary):ti,ab,kw |
| #6MeSH descriptor: [Pulmonary Infarction] explode all trees |
| #7(Pulmonary Infarctions):ti,ab,kw OR (Infarction*, Pulmonary):ti,ab,kw |
| #8 MeSH descriptor: [Venous Thromboembolism] explode all trees |
| #9 (Thromboembolism, Venous):ti,ab,kw |
| #10 MeSH descriptor: [Venous Thrombosis] explode all trees |
| #11 (Thrombos?s, Venous):ti,ab,kw OR (Phlebothrombos?s):ti,ab,kw OR (Deep-Venous Thrombos?s):ti,ab,kw OR (Thrombos?s, Deep Venous):ti,ab,kw OR (Deep Vein Thrombos?s):ti,ab,kw |
| #12 (Deep Venous Thrombos?s):ti,ab,kw OR (Deep-Vein Thrombos?s):ti,ab,kw OR (Venous Thrombos?s, Deep):ti,ab,kw OR (Thrombos?s, Deep-Venous):ti,ab,kw OR (Thrombos?s, Deep-Vein):ti,ab,kw |
| #13 (Vein Thrombos?s, Deep):ti,ab,kw OR (Thrombos?s, Deep Vein):ti,ab,kw |
| #14 (prevalence):ti,ab,kw OR (incidence):ti,ab,kw OR (occurrence):ti,ab,kw OR (incidence rate):ti,ab,kw OR (occurrence rate):ti,ab,kw |
| #15 #1 OR #2 OR #3 |
| #16 #4 OR #5 OR #6 OR #7 OR #8 OR #9 OR #10 OR #11 OR #12 OR #13 |
| #17 #14 AND #15 AND #16 |

**Table 4 Search Strategies of PubMed**

| ((((((((pulmonary disease, chronic obstructive[MeSH Terms]) OR lung diseases, obstructive[MeSH Terms]) OR Bronchitis, Chronic[MeSH Terms]) OR ((((((COPD[Title/Abstract]) OR COAD[Title/Abstract]) OR Chronic Obstructive Pulmonary Disease[Title/Abstract]) OR Chronic Obstructive Airway Disease[Title/Abstract]) OR Chronic Obstructive Lung Disease[Title/Abstract]) OR Chronic Airflow Obstruction*[Title/Abstract]) OR ((Acute exacerbation of chronic obstructive pulmonary disease[Title/Abstract]) OR AECOPD[Title/Abstract]))) AND ((((((Pulmonary Embolism[MeSH Terms]) OR Pulmonary Infarction[MeSH Terms]) OR ((Pulmonary Embolism*[Title/Abstract]) OR Pulmonary Thromboembolism*[Title/Abstract]))) OR Venous Thromboembolism[MeSH Terms]) OR Venous Thrombosis[MeSH Terms]) OR (((((((((((Phlebothrombos*[Title/Abstract]) OR Thrombos*, Venous[Title/Abstract]) OR Venous Thrombose[Title/Abstract]) OR Deep Vein Thrombos*[Title/Abstract]) OR Thromboses, Deep Vein[Title/Abstract]) OR Vein Thrombos*, Deep[Title/Abstract]) OR Deep-Venous Thrombos*[Title/Abstract]) OR Thrombos*, Deep-Venous[Title/Abstract]) OR Deep-Vein Thrombos*[Title/Abstract]) OR Thrombos*, Deep-Vein[Title/Abstract]) OR Deep Venous Thrombos*[Title/Abstract]))) AND (((((prevalence[Title/Abstract]) OR incidence[Title/Abstract]) OR incidence rate[Title/Abstract]) OR occurrence[Title/Abstract]) OR occurrence rate[Title/Abstract])) |
| --- |

**Table 5 Risk of bias for included studies**

| **Study** | **Item 1** | **Item 2** | **Item 3** | **Item 4** | **Item 5** | **Item 6** | **Item 7** | **Item 8** | **Item 9** | **Item 10** | **Overall Risk**  **Assessment** |
| --- | --- | --- | --- | --- | --- | --- | --- | --- | --- | --- | --- |
| Pek et al 2001 | N | N | N | Y | Y | Y | Y | Y | Y | Y | 7 |
| Akgun et al 2006 | N | N | N | Y | Y | Y | Y | Y | Y | N | 6 |
| Tillie-Leblondet al 2006 | N | N | N | Y | Y | N | Y | Y | Y | Y | 6 |
| Rutschmann et al 2007 | N | N | Y | Y | N | Y | Y | Y | Y | Y | 7 |
| Lessiani et al 2008 | N | N | N | Y | Y | Y | Y | Y | Y | Y | 7 |
| Gunen et al 2010 | N | N | N | N | Y | N | Y | Y | Y | Y | 5 |
| Duan et al 2010 | N | N | N | Y | Y | Y | Y | Y | Y | N | 6 |
| Dutt et al 2011 | N | N | N | Y | N | Y | Y | Y | Y | Y | 6 |
| Wang et al 2012 | N | N | N | Y | Y | N | Y | Y | Y | Y | 6 |
| Choi et al 2013 | N | N | N | Y | Y | Y | Y | Y | Y | Y | 7 |
| Kamel et al 2013 | N | N | Y | Y | Y | N | Y | Y | Y | Y | 7 |
| Liang et al 2013 | N | N | N | Y | Y | N | Y | Y | Y | Y | 6 |
| Akpinar et al 2014 | N | N | N | Y | Y | Y | Y | Y | Y | Y | 7 |
| Shapira-Rootman et al 2015 | N | N | N | Y | Y | Y | Y | Y | Y | Y | 7 |
| Bahloul et al 2015 | N | N | N | Y | N | Y | Y | N | Y | Y | 5 |
| Davoodi et al 2018 | N | N | Y | Y | N | Y | Y | Y | Y | Y | 7 |
| Pang et al 2018 | N | N | N | Y | Y | Y | Y | Y | Y | Y | 7 |
| Hassen et al 2019 | N | N | N | Y | N | Y | Y | Y | Y | Y | 6 |
| Dentali et al 2020 | N | N | N | Y | Y | N | Y | Y | Y | Y | 6 |
| Couturaud et al 2021 | N | N | Y | Y | Y | Y | Y | Y | Y | Y | 8 |

1. Was the study’s target population a close representation of the national population in relation to relevant variables, e.g., age, sex, occupation?
2. Was the sampling frame a true or close representation of the target population?
3. Was some form of random selection used to select the sample, OR, was a census undertaken?
4. Was the likelihood of non-response bias minimal?
5. Were data collected directly from the subjects (as opposed to a proxy)?
6. Was an acceptable case definition used in the study?
7. Was the study instrument that measured the parameter of interest (e.g.prevalence of low back pain) shown to have reliability and validity (if necessary)?
8. Was the same mode of data collection used for all subjects?
9. Was the length of the shortest prevalence period for the parameter of interest appropriate?
10. Were the numerator(s) and denominator(s) for the parameter of interest appropriate?

11. Summary item on the overall risk of study bias.

# Supplementary Figures


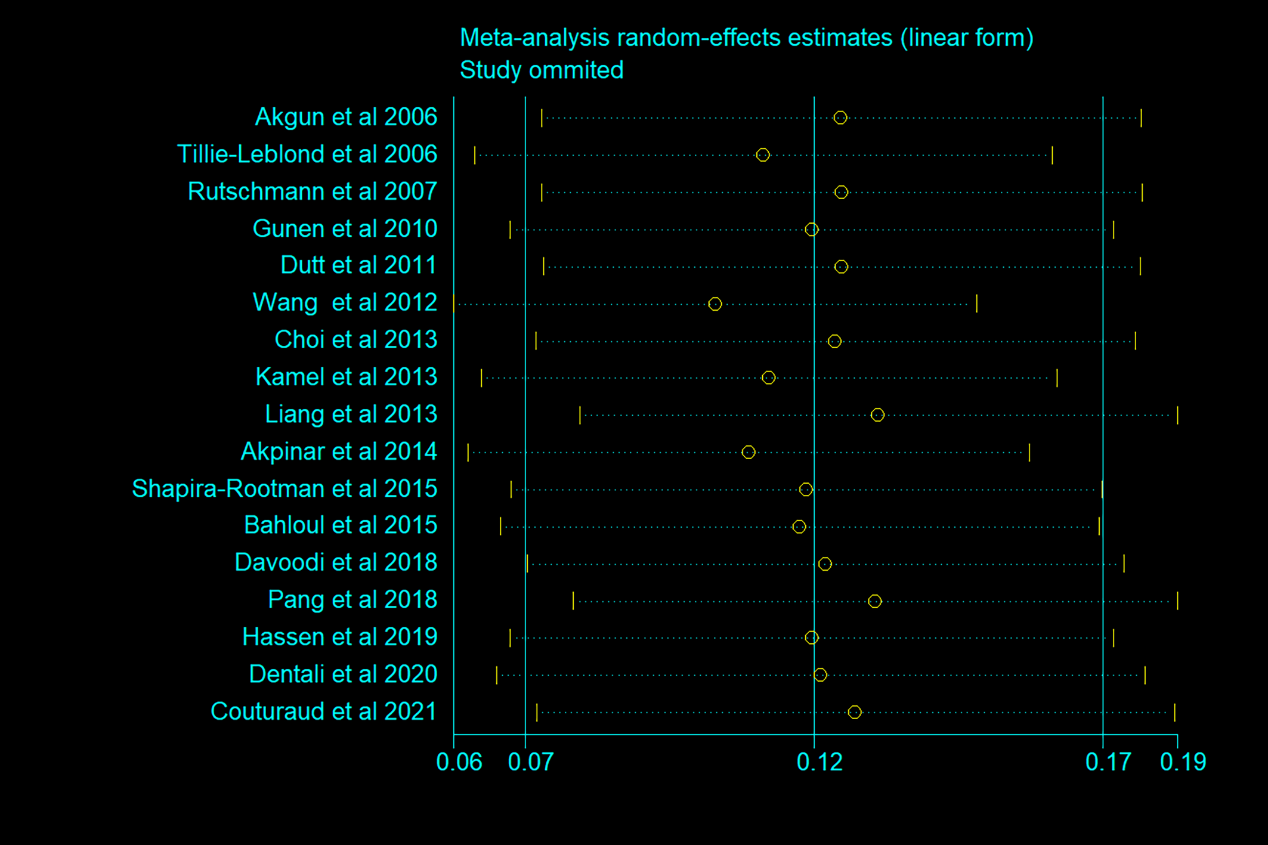


**Supplementary Figure 1. Sensitivity analysis of included studies about prevalence of PE in AECOPD**


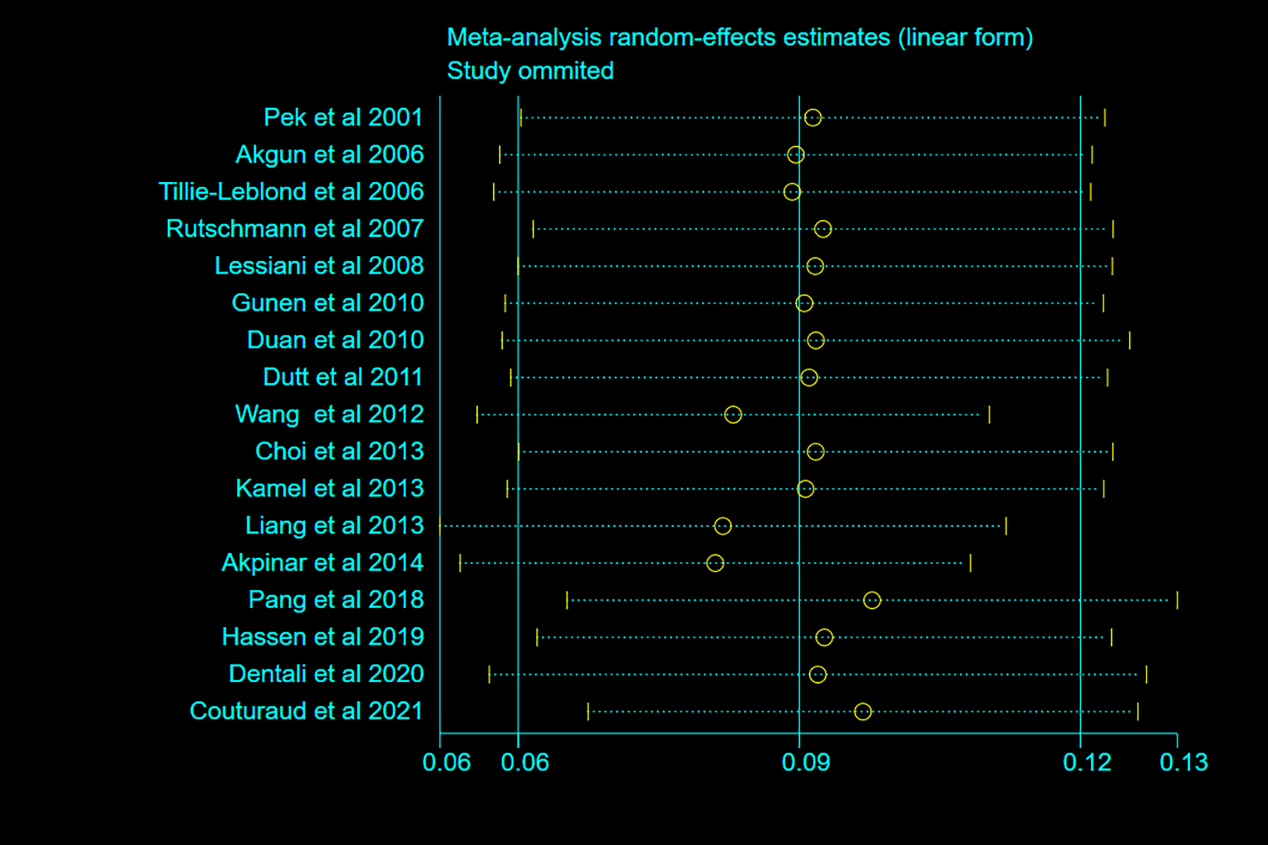


**Supplementary Figure 2. Sensitivity analysis of included studies about prevalence of DVT in AECOPD**


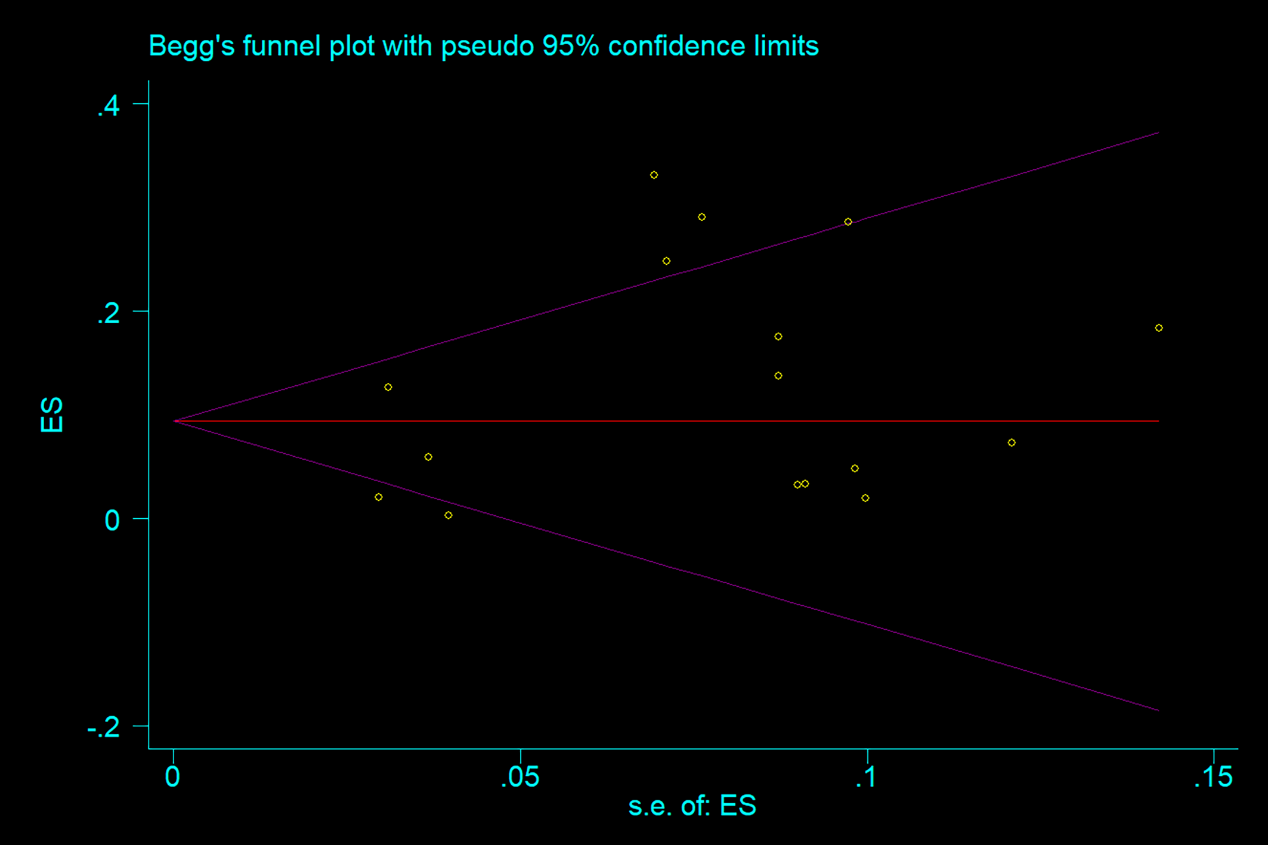


**Supplementary Figure 3. Begg’s funnel plot of included studies about prevalence of PE in AECOPD （Pr > |z| =1.000）**


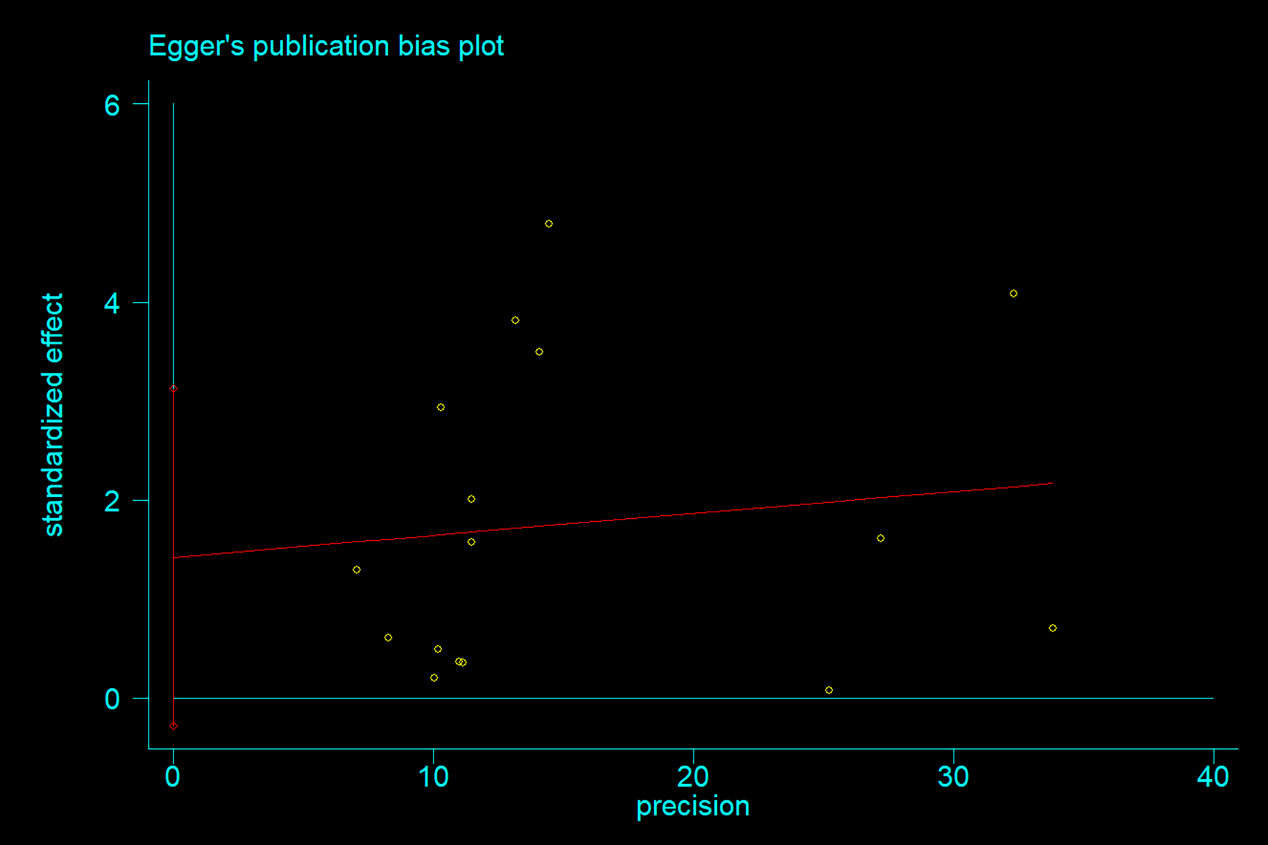


**Supplementary Figure 4. Egger’s publication bias plot of included studies about prevalence of PE in AECOPD （*P*=0.095，95%CI：-.2802483~3.129035）**


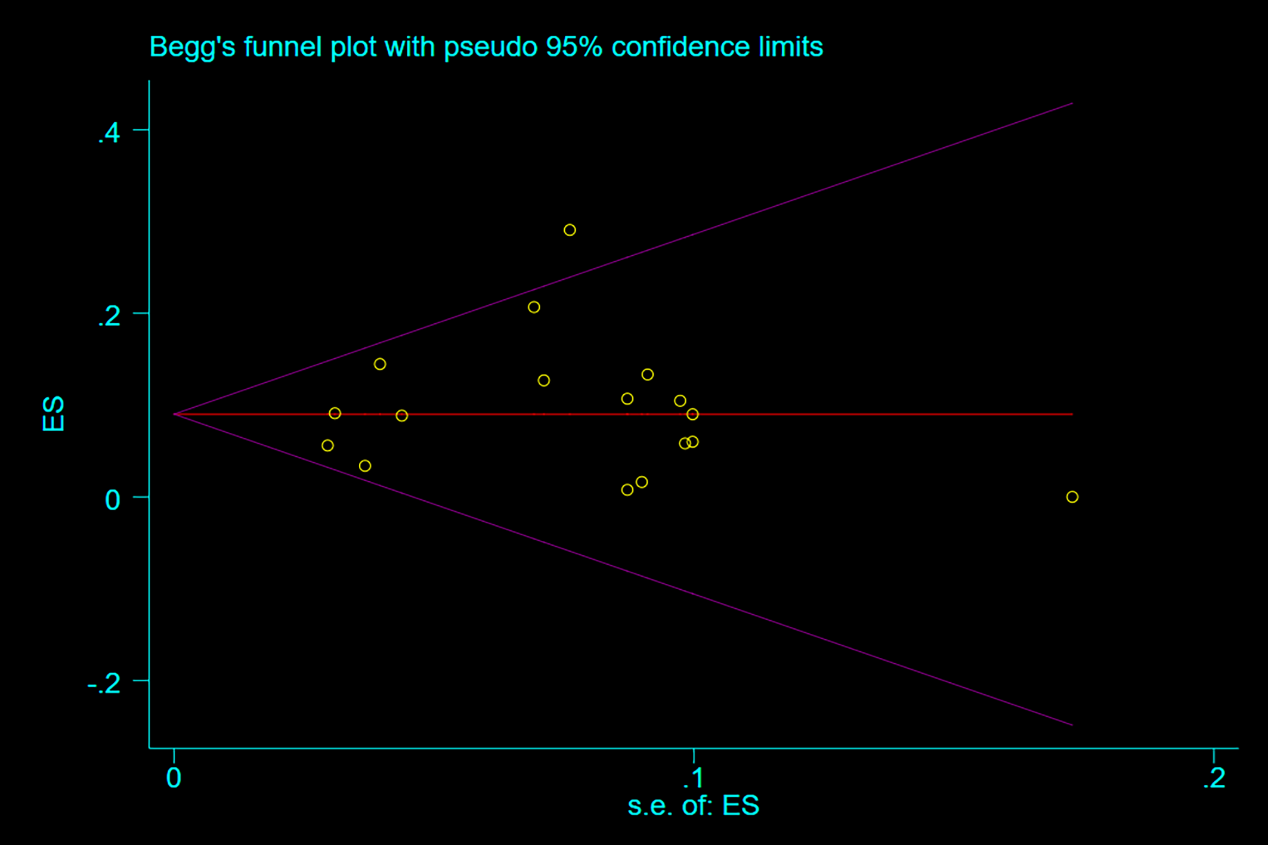


**Supplementary Figure 5. Begg’s funnel plot of prevalence of DVT in AECOPD（Pr > |z| = 0.773）**


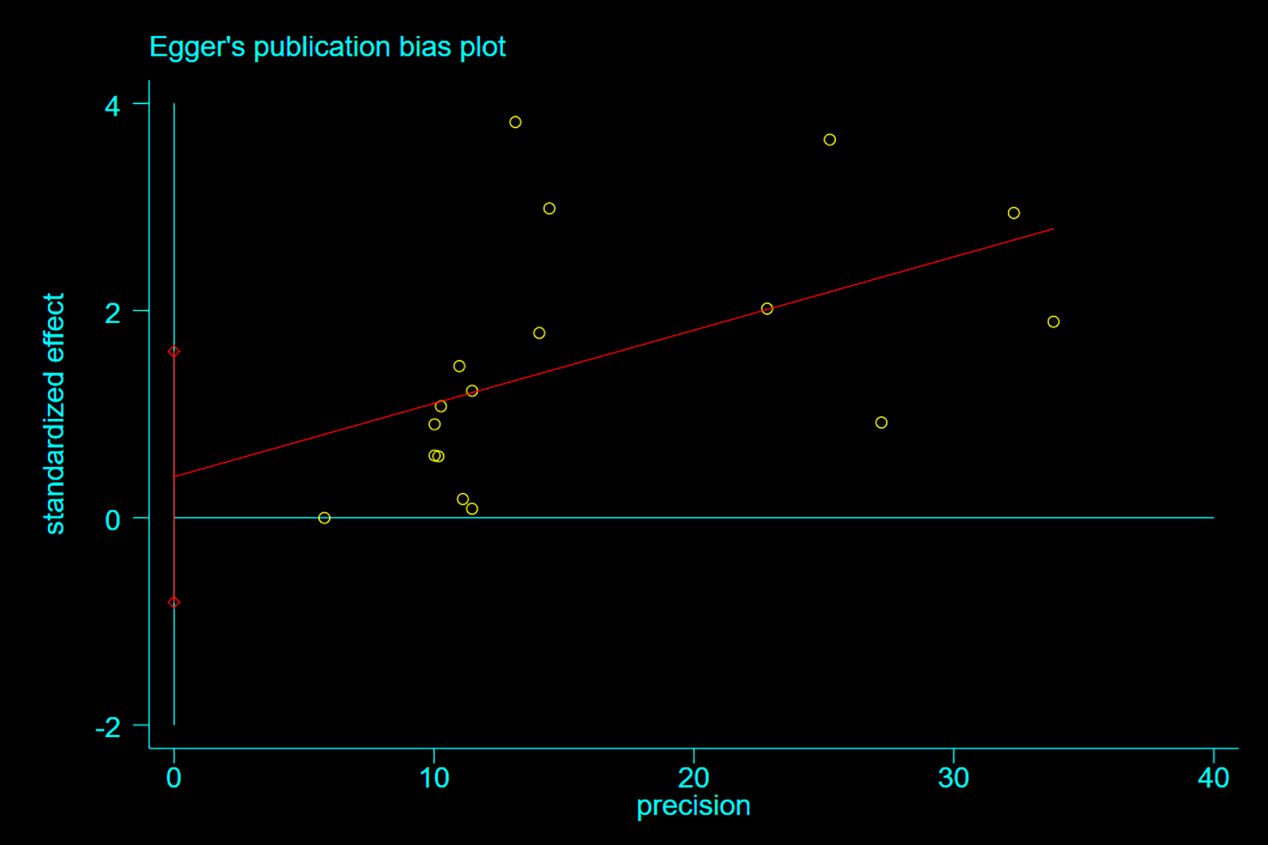


**Supplementary Figure 6. Egger’s publication bias plot of DVT in AECOPD**

**（*P*=0.496，95%CI：-.8137681~1.605933）**
